# Characterization of the Limbal Epithelial Stem Cell Niche

**DOI:** 10.1167/iovs.64.13.48

**Published:** 2023-10-31

**Authors:** Isabel Y. Moreno, Arian Parsaie, Tarsis F. Gesteira, Vivien J. Coulson-Thomas

**Affiliations:** 1 College of Optometry, University of Houston, Houston, Texas, United States; 2 College of Natural Science and Mathematics, University of Houston, Houston, Texas, United States

**Keywords:** heavy chain 5 (ITIHC5), inter-alpha-inhibitor, hyaluronan, cornea, limbus, extracellular matrix, glycosaminoglycans, proteoglycans

## Abstract

**Purpose:**

Limbal epithelial stem cells (LESCs) reside within a LSC niche (LSCN). We recently identified that hyaluronan (HA) is a major constituent of the LSCN, and that HA is necessary for maintaining LESCs in the “stem cell” state, both in vitro and in vivo. Herein, we characterized the LSCN to identify key components of the HA-specific LSCN.

**Methods:**

The cornea and limbal rim were dissected from mouse corneas, subjected to mRNA extraction, and sequenced using a NextSeq 500 (Illumina) and data processed using CLC Genomics Workbench 20 (Qiagen) and the STRING database to identify key components of the LSCN. Their expression was confirmed by real-time PCR, Western blotting, and immunohistochemistry. Furthermore, the differential expression of key compounds in different corneal cell types were determined with single-cell RNA sequencing.

**Results:**

We identified that the hyaladherins inter-alpha-inhibitor (IαI), TSG-6 and versican are highly expressed in the limbus. Specifically, HA/HC complexes are present in the LSCN, in the stroma underlying the limbal epithelium, and surrounding the limbal vasculature. For IαI, heavy chains 5 and 2 (HC5 and HC2) were found to be the most highly expressed HCs in the mouse and human limbus and were associate with HA-forming HA/HC-specific matrices.

**Conclusions:**

The LSCN contains HA/HC complexes, which have been previously correlated with stem cell niches. The identification of HA/HC complexes in the LSCN could serve as a new therapeutic avenue for treating corneal pathology. Additionally, HA/HC complexes could be used as a substrate for culturing LESCs before LESC transplantation.

The cornea is the clear outermost layer of the eye and, as such, is continuously exposed to insults. The cornea has various structural and physiological properties that enable it to heal and remain transparent after injury. One important characteristic of the cornea is the presence of limbal epithelial stem cells (LESCs) in the annular transition zone between the cornea and conjunctiva.^1^ LESCs are unipotent stem cells that differentiate into transient amplifying cells and move into the cornea, continuously replenishing the corneal epithelium with new cells.^1^^,^^2^ LESCs are important for the long-term maintenance of the corneal epithelium and critical for repopulating the corneal epithelium after injury.^2^^–^^4^ In tissues, stem cells are located within specific anatomical compartments, stem cell niches, which provide a specialized environment capable of maintaining them in an undifferentiated and self-renewable state.^5^^–^^8^ A substantial body of work has demonstrated that a specific microenvironment exists surrounding the limbal epithelial stem cells, forming the limbal epithelial stem cell niche (LSCN).^5^^,^^9^^–^^12^ One of the most important components of the stem cell niche is the extracellular matrix (ECM). The ECM is the noncellular component of tissues that provides essential structural support, serves as a reservoir for binding various growth factors, cytokines, and metalloproteases, and is responsible for providing various cues during development, homeostasis, and pathology.^13^^–^^16^

Each tissue contains an ECM with a unique composition that is capable of providing an optimal microenvironment necessary for carrying out its necessary functions. For example, cartilage is composed of proteoglycans (PGs), hyaluronan (HA), and collagen that form a resilient, resistant, and load-bearing tissue.^17^ The high glycosaminoglycan (GAG) content of the ECM in cartilage means it is a heavily hydrated tissue, which allows it to resist heavy impacts and also allows the free movement of molecules throughout the matrix of the non-vascularized tissue.^17^ ECMs are in a constant state of turnover, having the ability to rapidly respond to environmental stimuli and change as needed. This is evident after injury, where a provisional ECM is deposited to support the process of wound healing. Subsequently, this provisional ECM is reorganized into an ECM that can support normal tissue functions, as part of the process of regeneration.^18^ Importantly, the composition of the ECM that is present in the LSCN is distinct from that present in the peripheral and central cornea, and, also from that in the conjunctiva.^19^^,^^20^ Our group has recently demonstrated that HA-rich ECM exists within the LSCN, and that this HA matrix is necessary for maintaining LESCs in the “stem cell” state. This HA-matrix within the limbus assumes a net-like distribution surrounding the LESCs and forms “cable-like” structures, as we have previously shown.^5^^,^^19^^,^^21^^,^^22^ These “cable-like” structures run around the circumference of the limbus and extend into the peripheral cornea.^5^

HA is a linear polymer formed of a repeating disaccharide unit composed of glucuronic acid and N-acetylglucosamine and is one of the major components in tissues accounting for approximately 3% of the human dry body weight.^23^ Studies have shown that primarily two forms of HA exist in tissues: a high molecular weight HA (HMWHA) of approximately 2000 kDa and a low molecular weight HA (LMWHA) of approximately 200 kDa.^5^ HMWHA has anti-inflammatory effects and is primarily correlated with tissue integrity and is the form of HA that has been identified in stem cell niches, while LMWHA has pro-inflammatory effects and is primarily correlated with pathogenesis.^24^^–^^28^ Although HA is a structurally simple molecule, it interacts and binds to specific groups of proteins to form complex macromolecular matrices which regulate major physiological functions.^29^^–^^32^ For example, tumor necrosis factor (TNF)-stimulated gene-6 (TSG-6), a 35 kDa protein that is secreted by a wide range of cell types in response to inflammatory mediators, contains a HA link module domain through which it can bind and associate with HA.^33^ Inter-α-inhibitor (IαI, also known as ITI), is formed of a protein named bikunin that is covalently bound to a chondroitin sulfate chain that carries heavy chains (HCs). When HA, TSG-6, and IαI come in contact, TSG-6 catalyzes the transfer of HCs from IαI onto HA, forming an HA/HC/TSG-6 matrix.^30^ This culminates in the formation of a dense highly organized matrix with specific physiological functions. To date five different HCs have been identified that are capable of binding chondroitin sulfate (CS), namely HC1, HC2, HC3, HC5, and HC6.^31^ Additionally, TSG-6 modulates interactions between HA and CD44, the main cell surface receptor of HA.^32^ HA is also known to form a specialized matrix composed of aggrecan, brevican, tenascin, and proteoglycan link protein (Hapln1-4), as seen in perineuronal nets.^22^^,^^34^ This matrix is responsible for synaptic stabilization, and its formation directly correlates with closure of the critical period of plasticity.^34^ In cartilage, the specialized HA matrix is composed of HA, aggrecan, and Hapln1, and plays a vital structural role as well as regulate chondrogenesis.^35^^,^^36^ The synovial fluid contains a large quantity of uncomplexed HMWHA, which is required for the characteristic viscosity of the synovial fluid. Over the past decade, many studies demonstrated that specific HA matrices are present surrounding various types of stem cells. For example, a specific HA matrix composed of HA, HCs, TSG-6, versican and PTX3 is present around human umbilical cord mesenchymal stem cells (UMSCs) and is necessary for successful cell engraftment.^21^ As mentioned above, an HA matrix is present within the LSCN, and this matrix is required for maintaining viable LESCs^2^ and regulating lymphangiogenesis.^19^

Although our group has recently shown that a specialized HA-rich matrix is present in the LESC niche that regulates LESC differentiation and lymphangiogenesis, the exact composition of this HA matrix remains unknown.^19^ Herein, we worked to characterize the HA-specific matrix that is present in the corneal limbus of mouse, human and porcine corneas. We first used RNA sequencing (RNAseq) to screen for HA associated molecules and other ECM components in the limbus and cornea, and thereafter carried out a comparative analysis to identify targets that were differentially expressed. The differential expression profiles of the identified targets were validated by real-time PCR. Furthermore, the presence of HA/HC complexes was identified by Western blotting and the distribution of these complexes within the limbus characterized by immunohistochemistry. Thereafter, the differential expression of key compounds in different corneal cell types were determined with single cell RNA sequencing.

## Methods

### Animal Maintenance

The C57BL/6J mouse line was originally obtained from the Jackson Laboratory (Stock number 000664) and maintained under an automatic 12-hour light-dark cycle at the Animal Facility of the University of Houston. All mouse samples were obtained from eight-week-old C57BL/6J mice. All animal-related experimental procedures and handling were previously approved by the Institutional Animal Care and Use Committee (IACUC) at the University of Houston under protocols 16-025 and 16-044. Animal care and use conformed to the ARVO statement for the Use of Animals in Ophthalmic and Vision Research.

### MRNA Extraction and Processing

Sixteen eyeballs were collected and the corneas immediately isolated. Under a dissecting microscope, an experienced surgeon separated the limbal rim from the central cornea and conjunctiva. The isolated limbal rims and the corneal buttons were pooled and stored at −80°C until processed for RNA extraction. RNA libraries were prepared and sequenced at the University of Houston Sequencing and Gene Editing Core per standard protocols. Total RNA libraries were prepared with the QIAseq Stranded Total RNA kit (Qiagen, Hilden, Germany) using 100 ng input RNA. RNA was fragmented, reverse transcribed into cDNA and ligated with sequence adaptors. The size selection for libraries was performed using SPRIselect beads (Beckman Coulter, Southfield, MI, USA). Library purity was analyzed using the DNA HS1000 tape on a Tapestation 4200 (Agilent, Santa Clara, CA, USA) and quantified with Qubit Fluorometer (Thermo Fisher, St. Louis, MO, USA). The prepared libraries were pooled and sequenced using a NextSeq 500 (Illumina, San Diego, CA, USA); generating ∼20 million 2 × 75 bp paired-end reads per sample.

### RNAseq Data Analysis

RNA-seq fastq data was processed using CLC Genomics Workbench 20 (Qiagen). Illumina sequencing adaptors were trimmed, and reads were mapped to the *Mus musculus* reference genome GRCm38 (mm10). Normalization of RNA-seq data was performed using trimmed mean of M-values. Top under- and overexpressed genes were selected by differential expression analysis. Genes of interest selected from the sequence alignment were functionally annotated using the Database for Annotation, Visualization, and Integrated Discovery (DAVID) version 6.8 (https://david.ncifcrf.gov/home.jsp), and categories were selected of those that were of interest, mainly from the Kyoto Encyclopedia of Genes and Genomes (KEGG) pathways (https://www.genome.jp/kegg/) Our analysis focused on ECM components, ECM-receptor interactions, focal adhesion complexes, the TGF-β signaling pathway, the BMP signaling pathway, toll-like receptor signaling pathways and Wnt signaling. We also included glycosaminoglycan biosynthetic enzymes and metalloproteases (MMPs) in the analysis of the ECM components. Heatmaps were generated using Heatmapper (http://www.heatmapper.ca).^37^ The transcriptome was deposited in NCBI's Gene Expression Omnibus^38^^,^^39^ and are accessible through GEO Series accession number GSE244412 (https://www.ncbi.nlm.nih.gov/geo/query/acc.cgi?acc=GSE244412).

### Construction of Protein-Protein Interaction (PPI) Network

A network was built to show relationships among proteins using STRING database (https://string-db.org/) 10.1093/nar/gky1131. A network of differentially expressed genes showing protein-protein interaction was constructed and visualized using Cytoscape 10.1101/gr.1239303 for Has2, HC5, and TSG-6. Cytoscape plugin MCODE 10.1101/gr.1239303 was used to extract key modules of hub genes by filtering the relevant networks with a degree cutoff of 10.

### Real-Time PCR

A total of 12 mouse limbal rims and corneal buttons were obtained, as outlined above, and processed for RNA extraction using Trizol Reagent (Invitrogen, Carlsbad, CA, USA). The total RNA concentration was estimated using the absorbance values at 260 nm and purity was estimated using the OD 260/280 nm absorbance ratio. First strand cDNA was reverse transcribed from 2 mg of total mRNA using the high-capacity cDNA Reverse Transcription kit (Catalog no. 4368814, lot 00593854; Applied Biosystems, Lithuania), according to the manufacturer's protocol. Quantitative real-time PCR (qPCR) was carried out using 50 ng of cDNA, specific primers (Supplementary Table 1) and PowerUp SYBR Green Master Mix (Catalog no. A25918; Applied Biosystems) in CXF Connect Realtime System from Bio-Rad Laboratories (Hercules, CA, USA) using the activation cycle of 95°C for 10 minutes, 40 cycles of 95°C for 15 seconds, and 60°C for one minute. The specificity of the amplified products was analyzed through dissociation curves generated by the equipment, yielding single peaks. Negative controls were used in parallel to confirm the absence of any form of contamination in the reaction. Analysis of the data was carried out using both the 2*^−^^Δ^^Ct^* and 2*^−^^ΔΔ^^Ct^* methods using the CXF Connect Real Time System software and Microsoft Office 10 excel. The expression levels of all genes were normalized using both β-actin and GAPDH as housekeeping controls.

### ECM Extraction

Five human donor corneas that were deemed unsuitable for transplantation were obtained from Saving Sight and Miracles for Sight. Pig corneas were obtained from Sioux-Preme Packing Company. On arrival, the human and pig corneas were dissected under a dissection microscope (Leica S9E; Leica, Wetzlar, Germany) to isolate the limbal rims and corneal buttons, and thereafter the isolated tissues were stored at −80°C. Tissues were minced in 4 M guanidinium chloride in 0.05 M sodium acetate (pH6) containing protease inhibitors (Sigma G4505-500G, Sigma 320099; Sigma-Aldrich Corp., St. Louis, MO, USA) with PMSF (Sigma 329-98-6; Sigma-Aldrich Corp.) and incubated overnight at 4°C under agitation. The next day, the buffer was changed to 7 M urea in 0.05 M sodium acetate using Sephadex G50 columns (Millipore Sigma GE17-0042-01; Millipore Sigma, Burlington, MA, USA) at 4°C. GAGs and PGs were purified from the crude extract using Q-Sepharose fast flow chromatography eluting the GAG/PG fraction with 1 M NaCl in 0.05 M sodium acetate containing 7 M urea. The GAG/PG fraction was then desalted with PD10 Sephadex G25 (General Electric 17011778; General Electric, Boston, MA, USA) chromatography columns and dried using a lyophilizer (Labconco 82019-038; Labconco Corporation, Kansas City, MO, USA). Samples were suspended in double distilled H_2_O and protein content assayed using the Pierce Rapid Gold BCA Protein Assay kit (GL100016; ThermoFisher, St. Louis, MO, USA).

### Western Blotting

ECM extracts obtained from each sample were digested or not with hyaluronidase extracted from *Streptomyces* overnight at 37°C (Millipore Sigma H1136-5 × 1AMP) to release proteins and PGs from the HA-specific matrices. Both digested and nondigested samples were analyzed in parallel by Western blotting, as described previously.^21^^,^^40^ Samples (25 µg) were separated on a gradient (4%–20%) MiniPROTEAN TGX Stain-Free precast gels (Bio-Rad) by SDS-PAGE under reducing or nonreducing conditions, and thereafter transferred by electrical current to Immun-Blot Low Fluorescence polyvinylidene difluoride membranes (Bio-Rad) using the Trans-Blot Turbo transfer system (Bio-Rad 1704150EDU). The membranes were blocked in 5% BSA and developed with rabbit anti-IαI (A0301, DAKO, kindly donated by the Cleveland Clinic) at 1:5000 dilution, followed by secondary donkey-anti-rabbit conjugated with Alexa Fluor 555 (at a 1:10,000 dilution. The membranes were imaged using the ChemiDoc MP imaging system (Bio-Rad Universal Hood III).

### Immunohistochemistry

Five mouse eyeballs, and human and porcine corneas, were obtained and immediately fixed in 4% buffered paraformaldehyde and embedded in Tissue-Tek embedding medium (Sakura Finetek USA, Inc., Torrance, CA, USA). Tissues were sectioned into 10 µm sections using a Leica CM 1950 cryostat and mounted onto Fisherbrand SuperfrostPlus Gold microscope slides (Thermo Fisher Scientific, Waltham, MA, USA). Upon use, sections were incubated on a slide warmer (Premiere XH-2002) for 30 minutes at 60°C, and excess tissue embedding medium was washed away with PBS. Tissues were blocked with 10% fetal bovine serum prepared in PBS containing 0.01 M saponin. Sections were then incubated with the primary antibodies against IαI (DAKO) at 1:100, HC5 (Santa Cruz SC-390885; Santa Cruz Biotechnology, Dallas, TX, USA) at 1:100, and HC5 (ThermoFisher PA5-24445) at 1:100, Phalloidin at 1:300, and a probe HA binding protein (Millipore, Burlington, MA, USA) at 1:500. Sections were then washed and incubated with the secondary antibody, conjugated with AlexaFluor 488 (Thermo Fisher Scientific) and NeutrAvidin (Thermo Fisher Scientific) conjugated with AlexaFluor 555 (Thermo Fisher Scientific) for one hour at room temperature. Secondary controls were carried out in parallel with the omission of the primary antibodies and did not yield any staining (results not shown). Slides were mounted in Fluoromount-G and imaged under an LSM 800 confocal microscope (Zeiss, Oberkochen, Germany).

### Analysis of Single-Cell mRNA Sequencing Data

Processed RNA-seq data from adult human corneas and from human corneas from developmental stages 10, 12 and 20–21 postconception weeks (PCW), were obtained from the University of California Santa Cruz (UCSC) cell browser (http://genome.ucsc.edu), 10.1093/nar/gkaa1070, under the corneal cell atlas project (http://retinalstemcellresearch.co.uk/CorneaCellAtlas) by Collin et al.^41^ A script to process the data from USCS and analyze the expression levels of HA associated proteins and small leucine-rich proteo-glycans is provided in the supplementary information (Supplementary Information Script). In short, the RNA matrix was further filtered in Scanpy (https://scanpy.readthedocs.io/en/stable/)^42^ to obtain gene expression profiles for marker genes per cluster, which were then used to construct dot plots. The presented dot plots summarize two types of information, the relative level of expression and the preponderance of cells within a cell population that express the target gene. Specifically, the color represents the mean expression within each cell cluster, and dot size indicates the fraction of cells in each category that expresses a gene.

### Statistical Analysis

All values are presented as means ± standard deviation of the mean. All analysis and quantifications were performed in a masked manner to avoid bias. Statistically significant differences were assessed by *t*-test or ANOVA, followed by post hoc test for multiple comparisons considering *P* ≤ 0.05 as statistically significant. Statistical analysis was carried out using GraphPad Prism version 5 software package (GraphPad Software, San Diego, CA, USA) and Microsoft Excel v16.53.

## Results

### Differential Expression of Genes Related to the ECM

**Figure 1.**
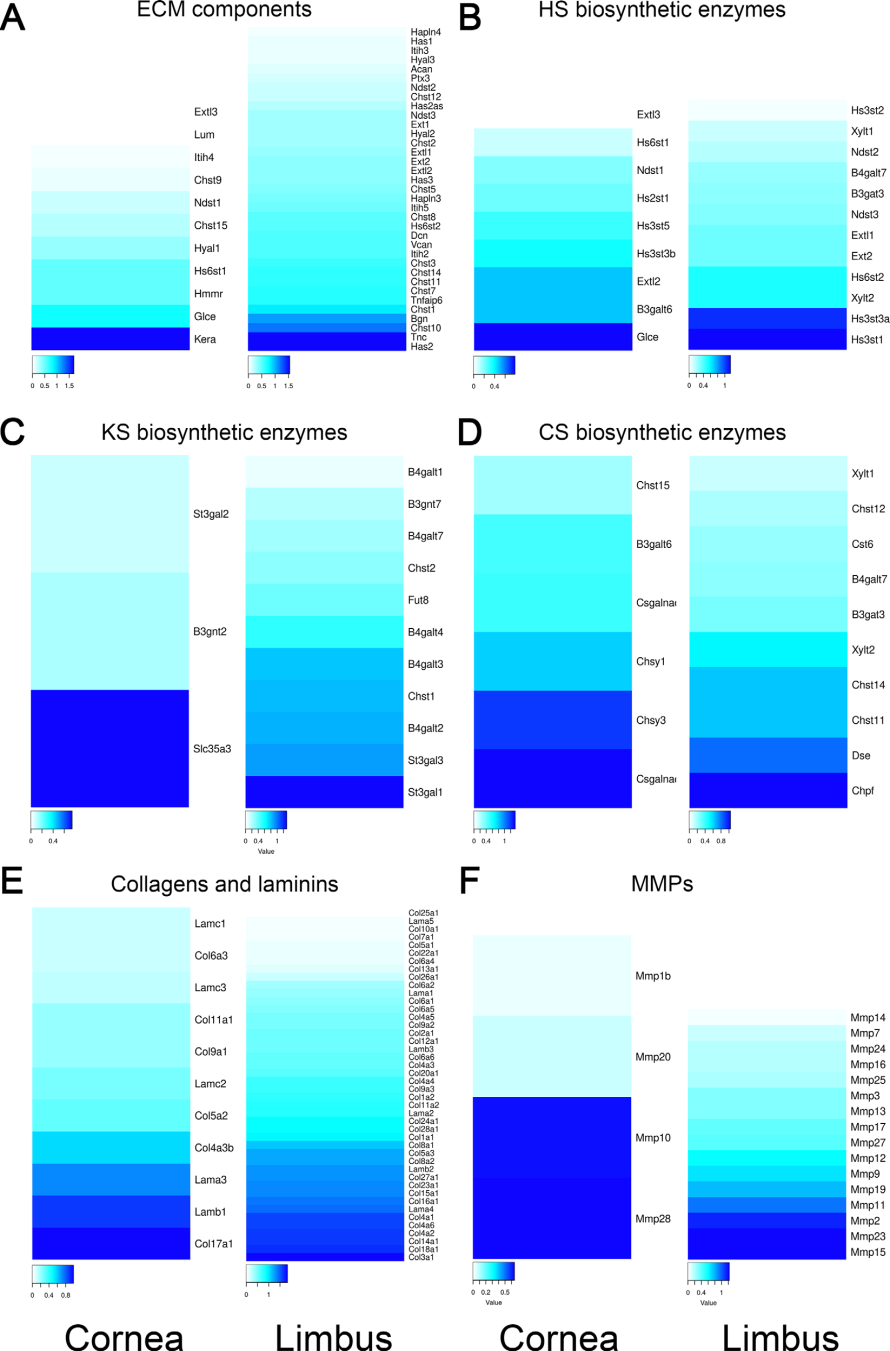
Differential expression of ECM related genes between the central cornea and limbus in mice. Heatmaps showing differential expression ECM components (**A**), heparan sulfate biosynthetic enzyme (**B**), keratan sulfate biosynthetic enzyme (**C**), chondroitin sulfate biosynthetic enzyme (**D**), collagen and laminin (**E**), and matrix metalloproteinase (**F**) genes between the central cornea versus the limbus. The heatmap representing the color-coded fold expression for each heatmap is shown underneath each heatmap. Darker blue colors represent a higher fold difference. Heatmaps to the left indicate genes that are upregulated in the central cornea when compared to the limbal region, and heatmaps to the right indicate genes that are upregulated in the limbus when compared to the central cornea.

**Figure 2.**
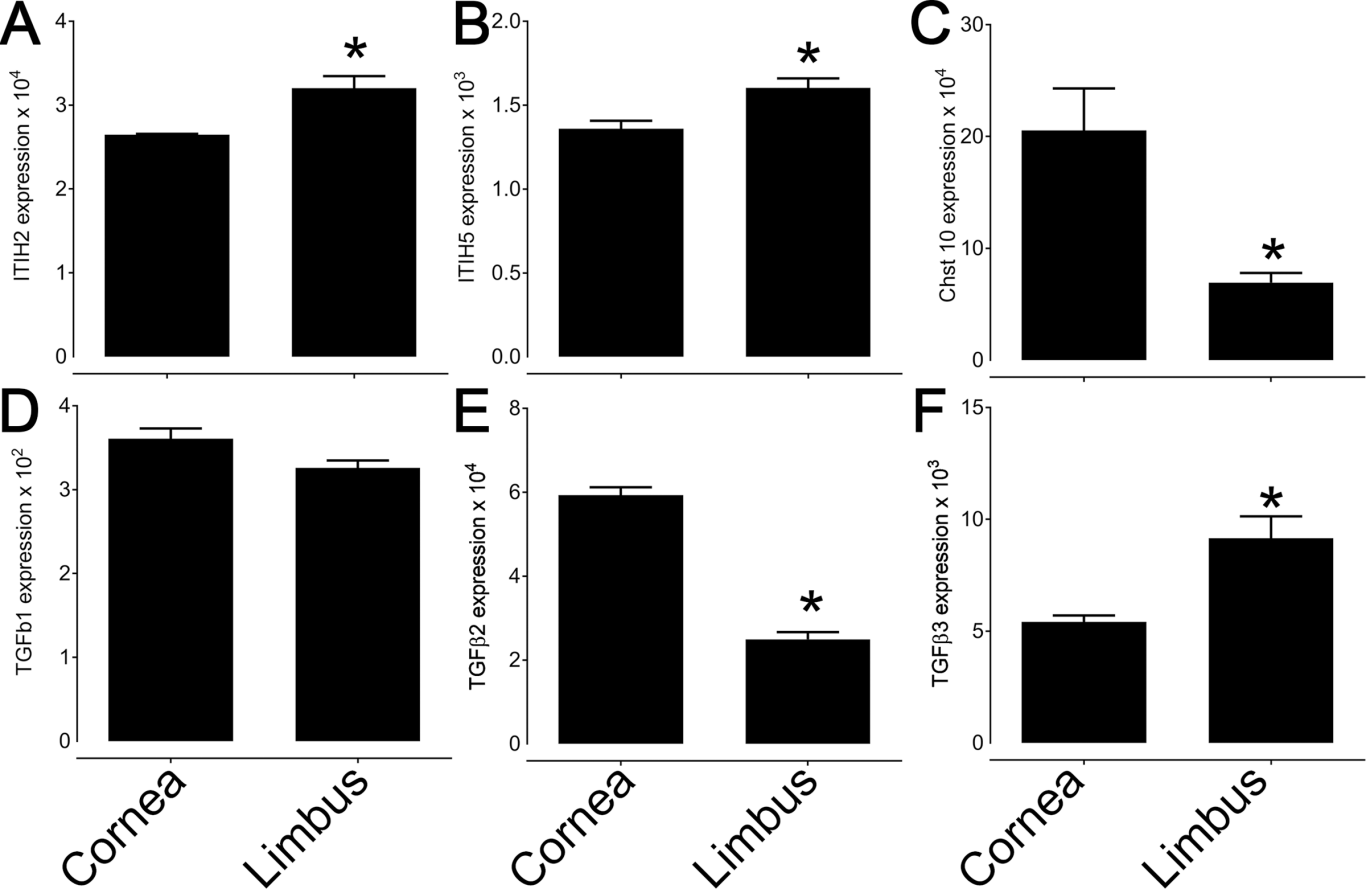
Confirmation of the differential expression of selected genes between the central cornea and limbus in mice. The differential expression of a selected number of genes, ITIH2 (**A**), ITIH5 (**B**), Chst10 (**C**), TGF-β1 (**D**), TGF-β2 (**E**), and TGF-β3 (**F**), between the cornea and limbus was confirmed by real time PCR. *Asterisk* represents *P* ≤ 0.05.

RNAseq analysis was carried out comparing the expression profile of various genes related to the ECM between the limbal region and cornea (Fig. 1). Interestingly, there was an increase in the expression levels of genes encoding the enzymes involved in the biosynthesis of HA, namely hyaluronan (Has) 1, 2 and 3, in the limbal region when compared to the cornea (Fig. 1A). Curiously, no significant changes in the expression levels of the hyaluronidases were noted between the limbus and the cornea. For protein and proteoglycans that are known to associate with HA, the expression levels of TSG-6, versican, IαI and tenascin were all found to be increased in the limbus when compared to the cornea (Fig. 1A). For IαI, the expression of heavy chains 1 to 5 were detected, although HCs 2 and 5 were the most highly expressed (Fig. 1A). We further confirmed the expression of HC2 and HC5 by qPCR and found that both were indeed expressed in the murine cornea and upregulated in the limbal region when compared to the central cornea (Figs. 2A, 2B).

We also analyzed the expression profiles of other ECM components. In terms of the small leucine-rich proteoglycans, decorin and biglycan were found to have increased expression in the limbus, whereas keratocan had increased expression in the cornea. No changes in the expression levels of lumican were noted between the limbus and central cornea. When the expression levels of genes related to biosynthesis of HS were analyzed, no striking changes were noted that would lead to significant changes in the levels and structure of HS between the limbal region and cornea (Fig. 1B). When genes related to the biosynthesis of keratan sulfate were analyzed, there was an increase in β-1,4-glucotransferases and carbohydrate sulfotransferases (Chst) 1 and 2 in the limbus (Fig. 1C). These enzymes are responsible for N-glycan and O-glycan biosynthesis, β-1,4-glucotransferases enable UDP-glucotransferase activity and Chst 1 and 2 catalyze the sulfation of keratan. When genes related to the biosynthesis of chondroitin sulfate (CS) were analyzed, there was a tendency toward an increase in the expression levels of the Chst genes in the limbus when compared to the cornea (Fig. 1D). More specifically, all Chst genes were increased in the limbus, with the exception of Chst15 (Fig. 1D). We also observed an overall increase in the expression of collagen in the limbus compared to the cornea and no significant differences in laminin expression (Fig. 1E). When analyzing the expression levels of the various MMPs, there was a significant trend toward an increase in MMP expression in the limbal region when compared to the cornea (Fig. 1F).

### Differential Expression of Genes Related to Signaling Pathways

The expression profile of genes related to signaling pathways that have previously been shown to regulate the expression and turnover of the ECM were analyzed. Specifically, TGF-β, Wnt, TLR, and BMP signaling pathways have been shown to be involved in synthesis and turnover of HA and HA specific matrices.^43^^,^^44^ Overall, our RNAseq data shows a tendency toward increased expression of members of the TGF-β signaling family in the limbus when compared to the cornea (Supplementary Fig. S1A). The expression levels of genes related to the TGF-β signaling were further confirmed by qPCR, demonstrating increased expression of TGF-β3 in the limbus when compared to the cornea (Figs. 2D–F). For members of the Wnt signaling pathway, there was a significantly increased expression of Wnt6 and Wnt5b, and a decreased expression of Wnt5a, Wnt3a, Wnt2 and Wnt9a in the limbus when compared to the cornea (Supplementary Fig. S1B). There was also an increased expression of Tlr12 and Tlr2 in the limbus, and decreased expression of Tlr4 in comparison to the cornea (Supplementary Fig. S1C). For members of the BMP signaling pathway, there was a significantly increased expression of BMP7, 1, 2 and 4, and a decreased expression of BMP3, 2k and 6 in the limbus when compared to the cornea (Supplementary Fig. S1D).

### Differential Expression of Keratins and LESC Markers Between the Limbal Region and Cornea

To ensure our samples were dissected appropriately, we also verified whether markers for LESCs were enriched in the limbal region and markers of corneal epithelial differentiation were enriched in the central cornea. As anticipated, the limbus presented higher expression levels of keratin 15, keratin 14, and keratin 19, whereas keratin 5 and keratin 12 were enriched in the central cornea (Supplementary Fig. S1F).

### Distribution of Differentially Expressed Genes Within PPI Networks of Has2 and HC5

**Figure 3.**
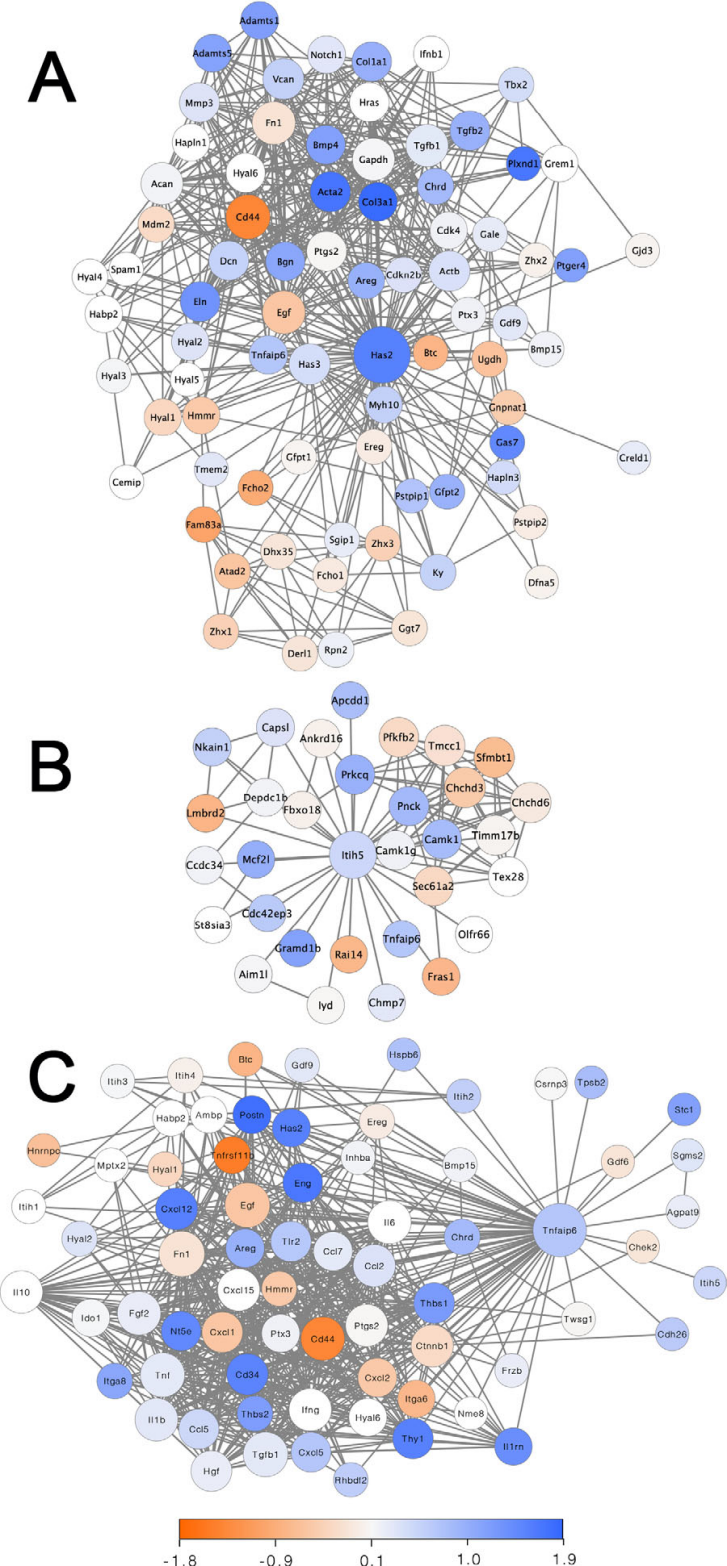
Protein-protein interaction (PPI) distribution networks for Has2, ITIH5 and TSG-6. PPI networks were built with the STRING database using Cytoscape for Has2, HC5, and TSG-6. The intensity and color indicate the fold change and whether the gene is upregulated (*blue*) or downregulated (*red*). The size of the circle indicates the number of interactions, where genes related with a high number of genes are represented with a larger circle.

In this study, we also constructed PPI networks to investigate the differential expression of genes related to HAS2 (Fig. 3A), HC5 (Fig. 3B), and TSG-6 (Fig. 3C). Based on the PPI network of differentially expressed genes, we found that most genes within the Has2 network were also upregulated within the limbal region when compared to the cornea, with the notable exception of the HA receptor CD44. We found most genes associated with the formation of HA specific matrices, for example Hapln1, Hapln3, tnfalp6 (gene encoding TSG-6) and Vcan, were significantly upregulated in the limbal region, while Acan and Ptx3 were not (Fig. 3A). Although limited research has been dedicated to understanding the interaction between HC5 and HA, within the PPI network for HC5, both HC5 and Tnfal6 were found to be more highly expressed in the limbal region (Figs. 3B, 3C).

### Identification of an HA/HC-Specific Matrix in the Limbal Region and Corneas by Western Blotting

**Figure 4.**
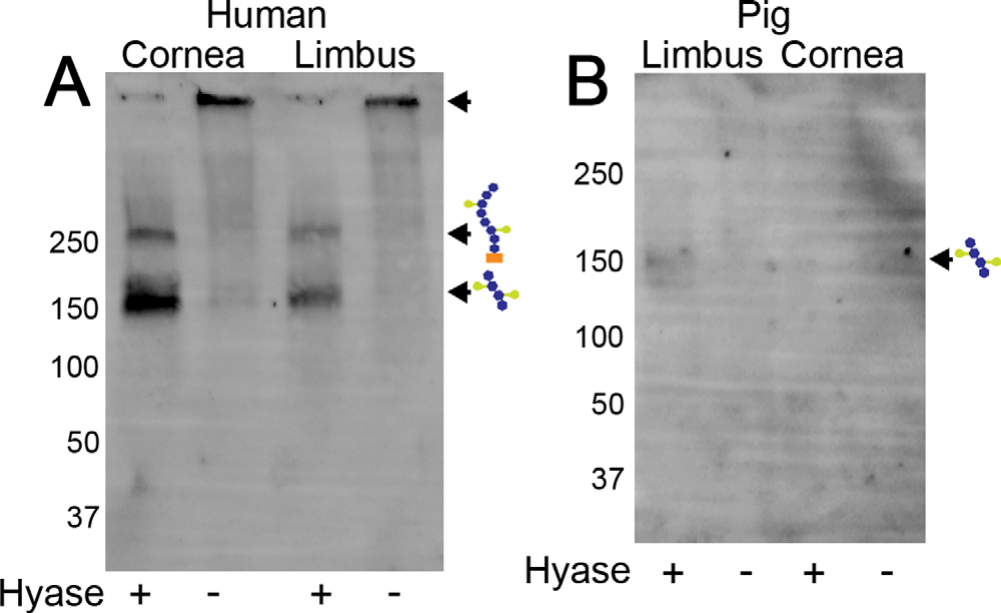
Identification of a HA/HC complex within the mouse, human, and porcine limbus and cornea by Western blotting. Western blot analysis of mouse (**A**), human (**B**), and porcine (**C**) central cornea and limbal crude extracts were digested with hyaluronidase or not and subjected to SDS-PAGE, transferred to polyvinylidene difluoride membranes and stained with anti-IαI (DAKO). Undigested samples show a large HA complex at the origin, whereas in digested samples, bands carrying IαI heavy chain fragments are able to migrate into the gel.

To confirm the presence of HCs in the LSCN of human and pig corneas, crude ECM extracts were obtained from human and pig limbal rim and corneal buttons. The crude ECM extracts were digested or not with hyaluronidase before analysis by Western blotting. Our data shows that a HA/HC specific matrix is present in both in the cornea and limbus of both human (Fig. 4A) and in the limbus of pig corneas (Fig. 4B). IαI was detected in a large HA complex that can be seen at the origin of the gel in samples that were not digested with hyaluronidase (lanes 2 and 4; Figs. 4A, 4B). However, upon hyaluronidase digestion the HA chain is cleaved, releasing the HA bound proteins and proteoglycans, and the HCs are able to enter the gel and can be seen as bands with the anti-IαI antibody (Figs. 4A, 4B). Based on the molecular weight of the complexes identified with the anti-IαI antibody, both intact IαI, IαI carrying only one HC, and a fragment of HA carrying two HCs were identified (Figs. 4A, 4B).

### Identification of an HA/HC-Specific Matrix in the Limbal Region and Corneas by Immunohistochemistry

**Figure 5.**
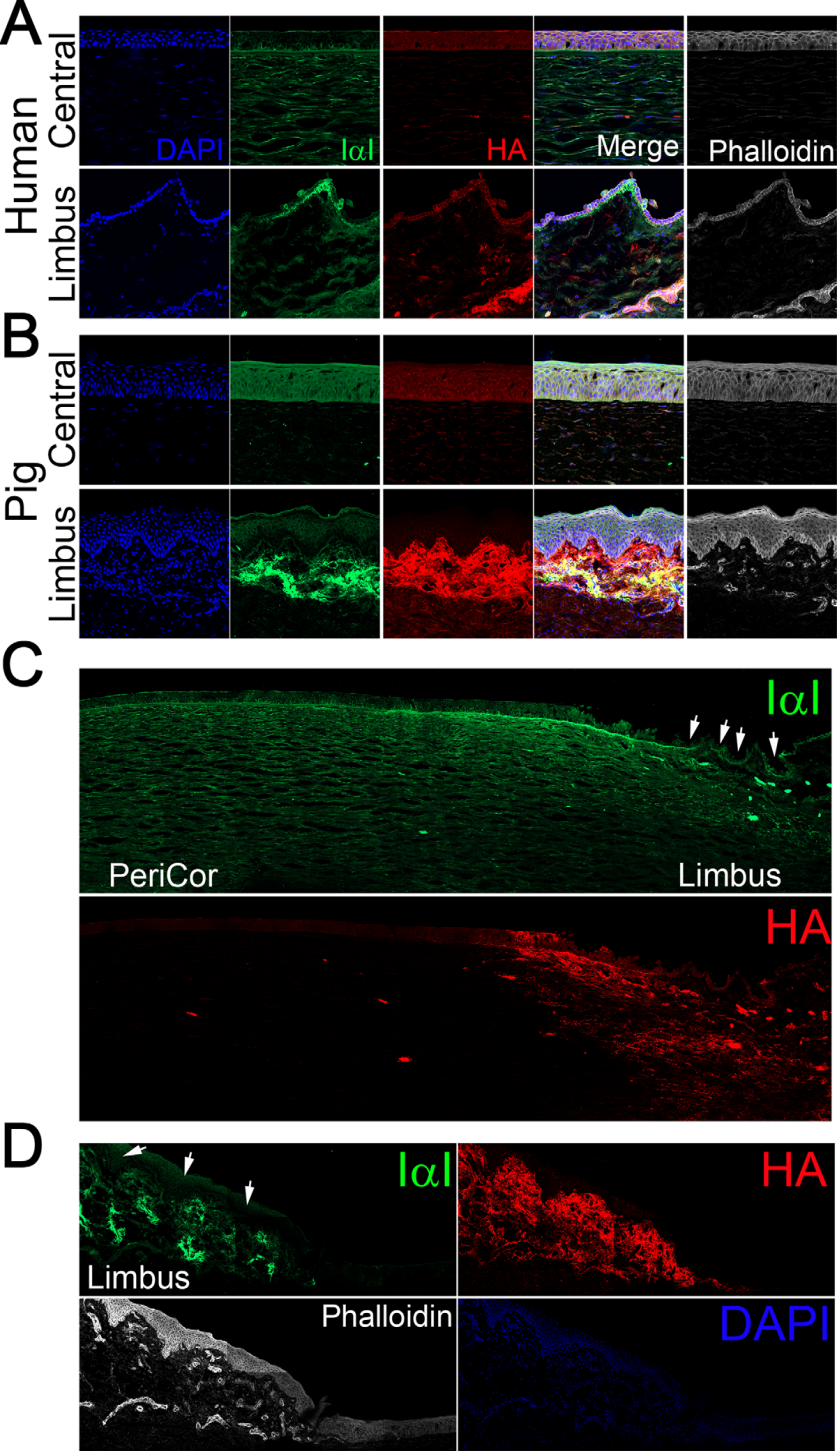
Characterization of the distribution of HA/HC specific matrices within the human and porcine corneas. Human (**A, C**) and porcine (**B, D**) corneas were fixed and prepared for immunohistochemical analysis of IαI (*green*) and HA (*red*). Nuclei were stained with DAPI.

To characterize the distribution of IαI within the cornea and limbal region, we carried out immunohistochemistry on human and porcine corneas. Anti-IαI from DAKO is a polyclonal antibody produced in rabbits against IαI from human serum. The antibody is highly specific and has been shown to recognize human and bovine bikunin, and mouse, rat, and human HCs.^45^^,^^46^ The specificity of the antibody has been validated in numerous studies over the years.^47^^–^^51^ In the human central cornea, we found that IαI is expressed at low levels in the epithelium, primarily in the basal lamina and basal layer of epithelial cells (Fig. 5A). In the human limbus, IαI is highly expressed in the palisades of Vogt, throughout all epithelial layers; however, it is most highly expressed in the basal layer (Fig. 5A). In the limbal region, IαI was also found to be expressed surrounding vessels and at lower levels throughout the stroma (Figs. 5A, 5C). In both the human corneal epithelium and limbal epithelium, IαI colocalized with HA, confirming that HCs participate in the formation of a HA/HC specific matrix in the human LSCN (Fig. 5A). In the pig corneas, IαI was localized in all epithelial layers in the central cornea and in the basal layer of the limbal epithelium (Fig. 5B). In the pig limbus, IαI was also expressed in the stroma underlying the epithelial crypts and surrounding vessels (Figs. 5B, 5D). HA colocalized with IαI in the basal layer of the central corneal epithelium, in the basal layer of the limbal epithelium, in the stroma underlying the limbal crypts and surrounding vessels in the limbal region (Fig. 5B).

### Identification of HC5 in the Limbal Region and Corneas by Immunohistochemistry

**Figure 6.**
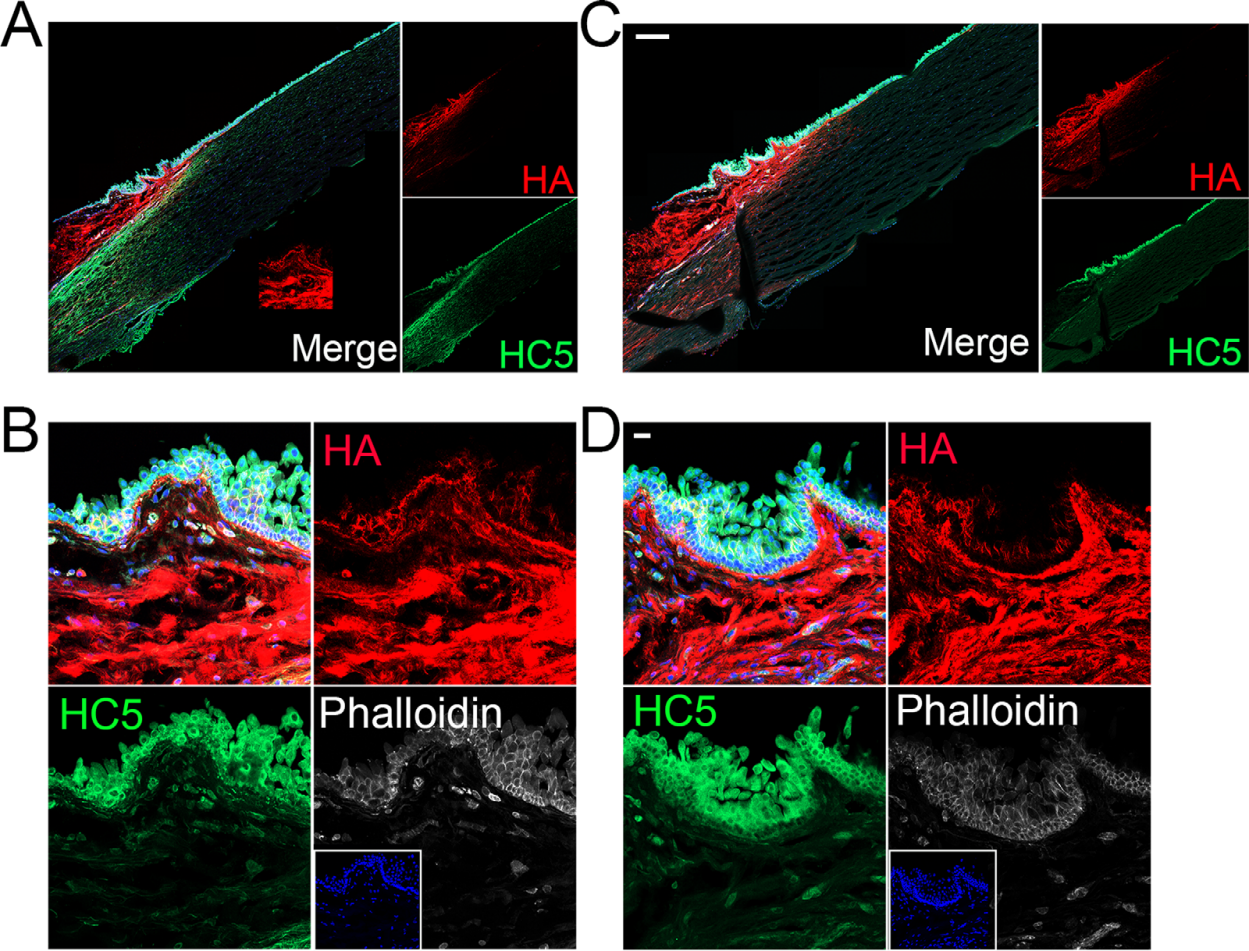
Characterization of the distribution of HC5 within the human corneas. Human corneas were fixed and stained with anti-HC5 (*green*) and HA (*red*). (**A, B**) Human corneas were stained with anti-HC5 by Santa Cruz (SC-390885), and (**C, D**) human corneas were stained with anti-HC5 by ThermoFisher (PA5-24445). The limbal region and peripheral cornea were imaged using the z-stack and tiling mode (**A, C**) or a single image was obtained of the limbal epithelium (**B, D**). Nuclei were stained with DAPI. *Scale bar:* 200 µm in **C** and 50 µm in **D**.

Immunohistochemistry was carried out on human corneas to characterize the distribution of HC5. Anti-HC5 staining achieved a similar tissue distribution to that of anti-IαI (Figs. 5, 6). Specifically, HC5 is highly expressed throughout all epithelial layers in the limbal region, with highest expression in the basal layer (Fig. 6). Importantly, HC5 staining colocalized with HA staining. HC5 was also found to be expressed in the epithelial layers in the peripheral cornea, surrounding vessels in the limbal region and in sparse cells throughout the limbal stroma, primarily in the anterior limbal stroma (Fig. 6).

### Expression of the HA-Related Matrix Throughout Different Cell Compartments Within the Developing and Adult Human Cornea by Single-Cell Sequencing

**Figure 7.**
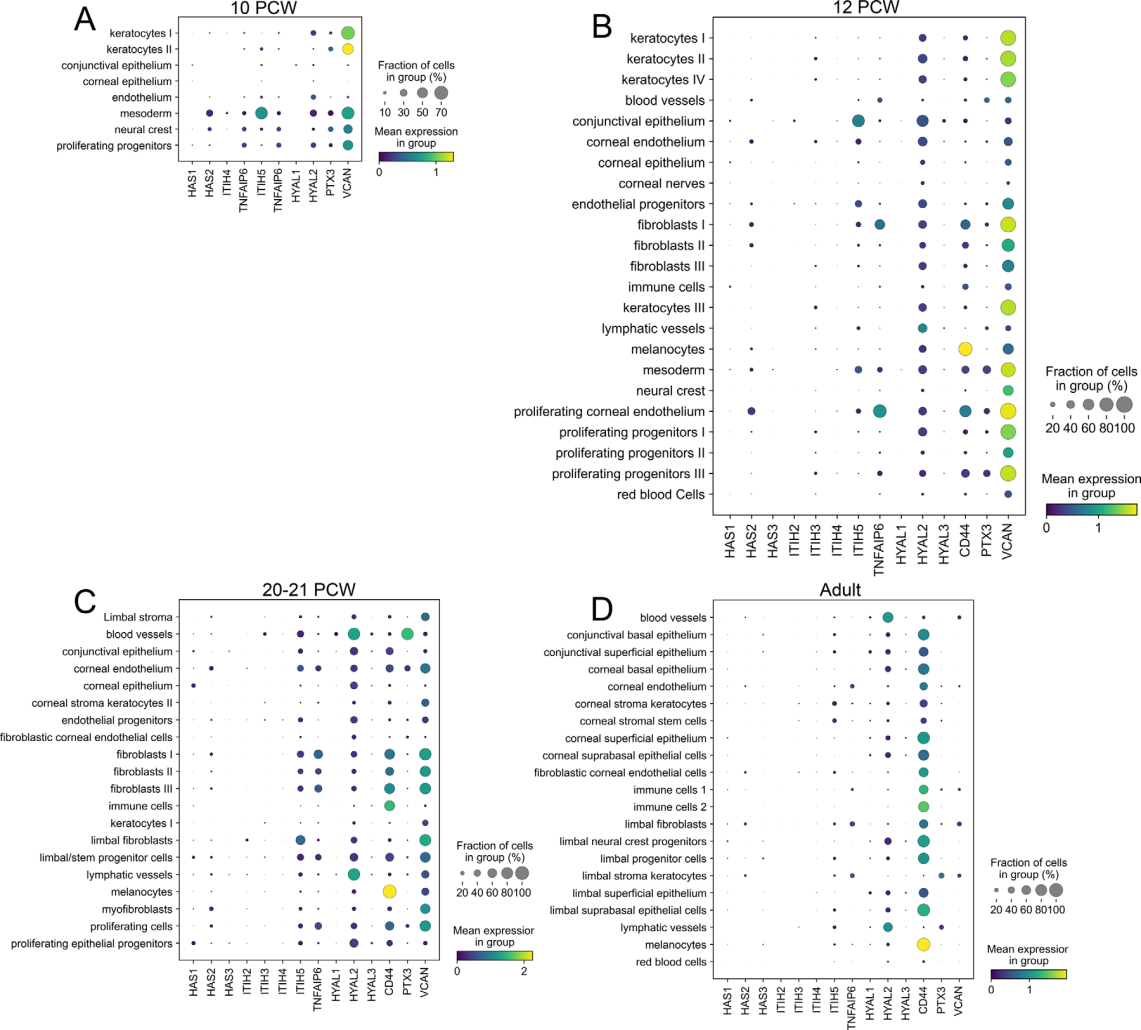
Single cell sequencing showing expression of HA-matrix related proteins in developing and adult human corneas. Mean expression of various HA-matrix related genes were analyzed in developing human corneas at 10 (**A**), 12 (**B**), and 20 to 21 (**C**) PCW and in adult corneas (**D**). The color represents the mean expression within each cell cluster, and dot size indicates the fraction of cells in each category that expresses a gene.

To assess the expression and distribution of HA throughout the human cornea, we investigated the expression profile of the HASs and HYALs by single-cell RNA sequencing, based on Collin et al.^41^ For the different HASs, we found that HAS1 is expressed at low levels by a small subset of cells (<20%) within almost all cell compartments (Fig. 7D). HAS2 is expressed by a subset (≤20%) of limbal stromal keratocytes, limbal fibroblasts, limbal progenitor cells, limbal neural crest progenitors, fibroblastic corneal endothelial cells, corneal stroma keratocytes, and corneal endothelium (Fig. 7D). HAS3 was found to be expressed at low levels by a small subset of cells (<20%) within the melanocyte, limbal suprabasal epithelial cell, limbal superficial epithelial cell, limbal progenitor cell, limbal neural crest progenitor cell, fibroblastic corneal endothelial cell, corneal subrabasal epithelial cell, corneal stromal stem cell, corneal stroma keratocyte, corneal endothelial cell and corneal basal epithelial cell populations (Fig. 7D). The expression levels of hyaluronidases were also investigated among the different cell populations of the adult human cornea, and we found that hyaluronidase type 2 (HYAL2) was the most highly expressed (Fig. 7D). HYAL2 was found to be primarily expressed in the cornea by lymphatic and blood vessels, with ∼80% of cells within these cell populations expressing HYAL2, followed by limbal neural crest progenitors, with ∼40% of cells expressing HYAL2 (Fig. 7D). Thereafter, all other cell populations also expressed HYAL2, although at lower levels and within ≤40% of cells (Fig. 7D). Therefore HYAL2 is expressed throughout the human cornea. HYAL 1 and 3 were also found to be expressed in the human cornea throughout most cell compartments, although at significantly lower levels than HYAL2 and by ≤20% of cells.

The expression profile of other LSCN molecules that associate with HA were also analyzed by single-cell RNA sequencing. For IαI, as seen with the mouse corneas, HC5 was the most highly expressed HC isoform in the human cornea (Fig. 7). ITIH5 was found to be expressed by ≤40% of cells within corneal stromal keratocytes and corneal stromal stem cells and by ≤20% of cells within melanocytes, lymphatic vessels, limbal suprabasal epithelial cells, limbal progenitor cells, limbal stroma keratocytes, limbal fibroblasts, fibroblastic corneal endothelial cells (Fig. 7D). ITIH5 was expressed by a smaller percentage of cells within all other corneal compartments (Fig. 7D). No ITIH1 and ITIH2 expression was detected in the human cornea, and negligible ITIH3 and ITIH4 expression was detected in a few corneal cell populations (Fig. 7D). TSG-6 was found to be expressed by ≤40% of cells within the limbal stromal keratocytes and limbal fibroblasts, and by ≤20% of cells within the corneal endothelium (Fig. 7D). PTX3 was found to be expressed by ≤40% of cells within the limbal stromal keratocytes and by ≤20% of cells within the lymphatic vessels (Fig. 7D). A very small amount of PTX3 was also found to be expressed by a small number of cells within all other cell compartments within the cornea (Fig. 7D). VCAN was also found to be expressed within the cornea by ≤40% of cells within the limbal fibroblasts, and ≤20% of cells within limbal stromal keratocytes, immune cells, and blood vessels (Fig. 7D). CD44, the main cell surface receptor for HA, was found to be highly expressed by ≥80% of cells throughout most corneal compartments (Fig. 7D). Specifically, CD44 was found to be highly expressed by ∼100% of melanocytes, limbal suprabasal epithelial cells, limbal neural crest progenitor cells, corneal superficial epithelium and corneal basal epithelium (Fig. 7D). Between 60–80% of cells within the limbal superficial epithelial cells, limbal progenitor cells and limbal neural crest progenitor cells also expressed CD44 (Fig. 7D). Thus most cells within the adult human limbal epithelium express CD44, via which these cells would bind to the HA matrix that is present within the LSCN.

The expression level of the different HA related proteins was also analyzed in different cell populations within the developing cornea (Figs. 7A–C). At 10 PCW, HAS2 is expressed primarily by mesodermal cells and neural crest cells, while at 12PCW it is primarily expressed by proliferating corneal endothelial cells, endothelial cells, and fibroblast populations, finally at 20–22 PCW, HAS2 is expressed at low levels in almost all corneal compartments (Figs. 7A–C). HAS1 and HAS3 are expressed at very low levels throughout most corneal compartments throughout all developmental stages analyzed (Figs. 7A–C). As seen in the adult cornea, HYAL2 is the primary HYAL expressed during development (Figs. 7A–C). At 10 PCW, all corneal compartments express HYAL2 in ≤30% of cells, and at 12 PCW, there is an increase in HYAL expression to ≤60% of cells (Figs. 7A–C). At 20 to 22 PCWs, HYAL continues to be expressed throughout all corneal cell compartments, with ∼100 % of cells within lymphatic and blood vessels expressing high levels of HYAL2 (Figs. 7A–C). HYAL 1 and 3 are also expressed within all corneal compartments, although by a very small subset of cells, ≤10% of cells (Figs. 7A–C). For IαI, ITIH5 is highly expressed in the mesoderm by ∼60% of cells and expressed at lower levels by ≤10% of cells in all other corneal cell compartments. ITIH4 was the only other HC detected at this stage and was found to be expressed by ≤10% of cells within the mesoderm, neural crest cells, and keratocytes I (Fig. 7A). By 12 and 20–21 PCW, ITI3, ITI4 and ITIH5 are expressed throughout all corneal compartments, although ITIH5 is the most significantly expressed (Figs. 7B, 7C). TSG-6 is expressed by ≤20% of cells within the mesoderm, neural crest, endothelium and keratocyte I cell compartments at 10 PCW, and by 12 PCW it is expressed by almost all corneal compartments, although most significantly by proliferating corneal endothelium (Figs. 7A, 7B). By 20–21 PCW, TSG-6 continues to be expressed by almost all corneal compartments, of note proliferating epithelial progenitors, limbal progenitor cells, and fibroblasts (Fig. 7C). Of all hyaladherins, VCAN was the most highly expressed throughout all developmental stages analyzed. At 10 PCW, VCAN was expressed by 40% to 70% of cells within the proliferating progenitor cells, mesoderm, neural crest cells and keratocytes. By 12 PCW, VCAN was expressed by all corneal cell types, primarily proliferating progenitors, neural crest cells, mesodermal cells, and fibroblasts (Fig. 7B). By 20–21 PCW, the number of cells expressing VCN has decreased, although all corneal cell compartments still contained cells that express VCAN (Fig. 7C). At this time point, primarily proliferating cells, myofibroblasts, limbal progenitor cells, limbal fibroblasts, fibroblasts, and the corneal endothelial cells express VCAN (Fig. 7C). PTX3 was expressed within most corneal cell compartments, although by ≤10% of cells. Notably, at 20–21 PCW ∼80% of cells within blood vessels expressed PTX3 (Fig. 7C).

## Discussion

In this study, we aimed to characterize the LSCN with the primary focus of identifying ECM components that are bound to HA in the cornea and limbal region. The limbal region was previously shown to contain an HA-specific matrix that is necessary for maintaining LESCs, and knock-out mice lacking enzymes involved in the biosynthesis of HA within the limbal region were shown to have a loss of LESCs.^52^ We went on to show that HA is present in the murine cornea primarily in the limbal epithelium, and assumes a net-like distribution surrounding the epithelial cells and forms cable like structures that extend into the cornea^52^ and that HMWHA can maintain LESCs during ex vivo expansion.^2^ Previous studies have demonstrated that HA associates with a number of proteins and proteoglycans to assemble HA-specific matrices.^30^^,^^33^^,^^53^^–^^56^ For example, in the central nervous system HA associates with aggrecan, link protein and tenascin to form specialized structures named perineuronal nets.^34^ HA has been shown to associate with HCs, TSG-6, pentraxin 3 and versican to form cable-like structures that surround umbilical cord mesenchymal cells (UMSCs).^48^^,^^57^ Thus we speculated that in the limbal region, HA must associate with hyaladherins enabling it to form a specific extracellular niche capable of supporting LESCs. Identifying the specific components of this niche will provide valuable information that can guide future studies into developing appropriate matrices for supporting LESCs both in vitro and in vivo.

Given our previous work demonstrated the HA-specific matrix was expressed primarily in the limbal region, we carried out an RNAseq analysis to identify genes that were differentially expressed between the limbal region and central cornea. We were able to identify that there was an increased expression of all HAS isoforms in the limbal region, when compared to the remaining cornea. This finding supports our previous study demonstrating that all three HAS isoforms are expressed in the limbus and cornea, and that HA is primarily expressed in the limbal region.^5^^,^^58^ We then actively searched the dataset for the expression levels of all proteins and proteoglycans known to associate or bind with HA. Of all those searched, IαI, TSG-6, versican, and tenascin were all found to be more highly expressed in the limbus when compared to the cornea. IαI is a PG that is composed of 3 polypeptides, a trypsin inhibitor named bikunin and two HCs, covalently bound to a CS.^59^ To date five HCs have been shown to exist and bind to HA.^45^^,^^54^^,^^60^ We hereby found that HCs 1 through 5 are expressed in the mouse limbal region, with HCs 2 and 5 being the most highly expressed. IαI was first identified in serum, and for many years was believed to be exclusively expressed in the liver.^60^^–^^62^ Since then, studies have identified various non-hepatic tissues that also express IαI, such as, adrenal, the appendix, bone marrow, brain, colon, duodenum, endometrium esophagus, fat, gall bladder, heart, lung, lymph node, ovary, pancreas, placenta, prostate, salivary gland, skin, small intestine, spleen, stomach, testis, thyroid, and urinary bladder,^46^^,^^63^^–^^65^ umbilical cord mesenchymal stem cells,^21^ the CNS^22^ and in certain cancers.^66^ We further confirmed the expression of HC2 and 5 in the cornea and limbal region of mouse corneas by real time PCR. We found that both HC2 and HC5 are indeed expressed in the cornea, with significantly higher expression levels in the limbal region. HC5 was also found to be the primary HC isoform expressed in the human cornea by single cell RNA sequencing. Previous studies have shown that the different HC isoforms have distinct biological functions.^63^^,^^67^^–^^70^ HC2 was first identified in human plasma and since then has been found to be expressed in the liver, central nervous system, ovaries, placenta, and testes in mice.^71^^–^^73^ HC5 was only recently identified and found to associate and bind to HA^74^ and has been found to be expressed primarily in fat tissue, placenta, and urinary bladder.^75^^–^^77^ To further confirm the expression of IαI in the cornea, and verify whether HCs are indeed transferred onto HA to form HA/HC matrices, we extracted the extracellular matrix from both human and pig corneas and subjected them to Western blotting analysis using a specific anti-IαI antibody. Interestingly, IαI was found to be expressed in the ECM of both the cornea and limbal region; however, it was identified as a large complex that was unable to migrate into a 4% to 12% gradient gel, indicating the HCs are bound to HA forming large macromolecules. When the samples were digested with hyaluronidase, the HCs were released and migrated at the molecular weight of 150 and 250 kDa. The 250 kDa band represents intact IαI, whereas the 150 kDa band represents two HCs.^78^ Immunohistochemistry further confirmed IαI and HC5 are expressed in the cornea limbus and colocalize with HA. Thus, based on our data, IαI is expressed in the cornea and limbal region, and HCs, in particular HC5, is transferred onto HA forming specialized HA/HC matrices. Interestingly, HC5 was also found to be the major HC expressed in the epidermis by Huth et al.^74^^,^^79^ This same group went on to show that in inflammatory skin diseases there is an increase in HC5 expression within the epidermis and inflammatory infiltrate.^74^^,^^79^

For more than two decades, the amniotic membrane, a tissue rich in HA/HC/TSG-6 complexes, has been used to promote wound healing, suppress inflammation and as an anti-scarring agent for ocular surface reconstruction.^80^^,^^81^ The amniotic membrane has also been extensively used as a substrate for culturing and transplanting LESCs onto the ocular surface, significantly augmenting transplantation success when treating LSCD.^82^^–^^85^ The Tseng group has successfully purified and characterized HA/HC/TSG-6 complexes from the amniotic membrane and show they are able to alone recapitulate the therapeutic benefits of the amniotic membrane.^86^ The Tseng group has also shown that the HA/HC complexes of the amniotic membrane are primarily made up of HMWHA covalently linked to HC1.^87^ Thus the amniotic membrane very closely recapitulates the ECM environment within LSCN, thus explaining why over the years the amniotic membrane has proven to be an optimal substrate for treating the ocular surface. However, the amniotic membrane has been shown to express solely HC1.^88^ Thus, based on our findings, HA/HC5 could offer improved therapeutic benefits over HA/HC1. Moreover, HA/HC5 could be used to support LESCs during ex vivo culture and as a potential new treatment strategy for LSCD with improved clinical outcome when compared to purified HA/HC1 and amniotic membrane.

In our study, TSG-6 was found to be expressed in the corneal limbal region at higher levels when compared to the remaining cornea. TSG-6 catalyzes the covalent transfer of HCs from IαI onto HA and can also bind and associate with HA, and, thus, TSG-6 plays a central role in the formation of HA/HC and HA/HC/TSG-6 specific matrices.^22^^,^^89^^–^^95^ The formation of HA/HC/TSG-6 complexes has been shown to have a role in most, if not all, inflammatory processes, such as promoting leukocyte adhesion to HA matrices.^48^^,^^50^^,^^96^ Whether the HA/HC matrices and TSG-6 identified in the cornea have a role regulating inflammation remains to be established. HA/HC/TSG-6 matrices have been shown to participate in the formation specialized niches surrounding various types of stem cells.^21^^,^^32^^,^^97^ We have previously shown that human umbilical cord mesenchymal stem cells synthesize an HA-specific matrix, namely a HA/HC/TSG-6/PTX3/versican matrix, that supports stem cell engraftment and enables these cells to survive xenograft rejection.^21^ Recently, TSG-6, HA and exogenous IαI were also shown to increase embryonic MSC engraftment into skeletal muscle and favor differentiation into muscle cells.^98^ Therefore TSG-6, HA, and IαI enable MSCs to generate a microenvironment that is important for embryonic MSC transplantation success.^98^

In this study, we also identified changes in the expression levels of various GAGs between the cornea and limbal region. We found an overall tendency of increased expression of CS biosynthetic enzymes in the limbal region when compared to the remaining cornea, indicating there could be increased levels of CS in the limbus. We previously characterized the structure of CS in the murine cornea, and found that after alkali burn there is an increase in highly sulfated forms of CS and a decrease in the lesser sulfate forms of CS.^20^ The expression profile of HS biosynthetic enzymes and HSPGs were also analyzed in this current study, and no differential expression was noted between the limbal region and remaining cornea. HS was previously shown to be necessary for maintaining the corneal and limbal epithelial integrity primarily by maintaining cell-cell and cell-matrix adhesion.^99^ We previously showed that HS expression levels and structure was not altered in the cornea after alkali burn.^20^ Special heterogeneity was previously demonstrated between the epithelial basement membrane of the human cornea, limbus, and conjunctiva.^100^ The central cornea epithelial basement membrane was shown to contain collagen IV composed of α3 and α5 chains, whereas the limbal epithelial basement membrane contains α1, α2 and α5 chains and laminin α2 and β2 chains.^101^ Additionally, the limbal region presents patchy distribution of laminin gamma3 chain, BM40/SPARC, and tenascin-C within the limbal palisades, which colocalize with cells expressing putative LESC markers.^102^ Furthermore, collagen XVI, fibulin-2, tenascin-C/R, vitronectin, bamacan, chondroitin sulfate, and versican are expressed at higher levels in the corneal-limbal transition zone colocalizing with clusters of cells believed to be late progenitor cells. Curiously, both of the latter studies noted that there were many similarities between the basement membrane of the limbal epithelium and conjunctival epithelium.

Finally, this study also analyzed the differential expression of signaling pathways previously shown to regulate the expression of ECM components during development, homeostasis and pathology. Worthy of note, members of the TGF-β signaling family were found to be more highly expressed in the limbal region, when compared to the remaining cornea. TGF-β signaling has been shown to regulate ECM deposition in various tissues, including the cornea.^103^^–^^105^ HA has also been shown to signal through TLRs, with HMWHA capable of suppressing TLR signaling while LMWHA promotes TLR signaling.^106^^,^^107^ In particular, LMWHA has been shown to signal through TLR2 and 4.^108^ Our study showed that TLR2 was primarily expressed in the limbus and thus could be involved in HA mediated signaling.

The cornea and limbal region carry out extremely different physiological functions.^109^^,^^110^ The limbal region supports the ingrowth and presence of blood and lymphatic vessels, contains numerous resident inflammatory cells, and supports two distinct stem cell pools, the limbal epithelial stem cells and the limbal mesenchymal stem cells.^9^^,^^111^ Additionally, the limbal region forms an important physical barrier between the conjunctiva and the cornea.^112^ In contrast, the primary physiological functions of the cornea are to protect the remaining components of the eye, provide the initial refractive power to light entering the eye and remain transparent.^113^ Taken together, this study demonstrates how the ECM in the cornea is significantly different in composition to that in the limbal region. These significant changes in the ECM composition throughout the cornea mean the ECM in these distinct compartments exert distinct signaling cues and have distinct biomechanical properties.

## Supplementary Material

Supplement 1

Supplement 2

Supplement 3
