# Supplementary material for: Characterization of the Limbal Epithelial Stem Cell Niche
Source: Invest Ophthalmol Vis Sci. 2023 Oct 31;64(13):48. doi: 10.1167/iovs.64.13.48 (PMC10619699; doi:10.1167/iovs.64.13.48)
Supplement: Supplement 1 [file iovs-64-13-48_s001.pdf]

Supplementary Figure 2

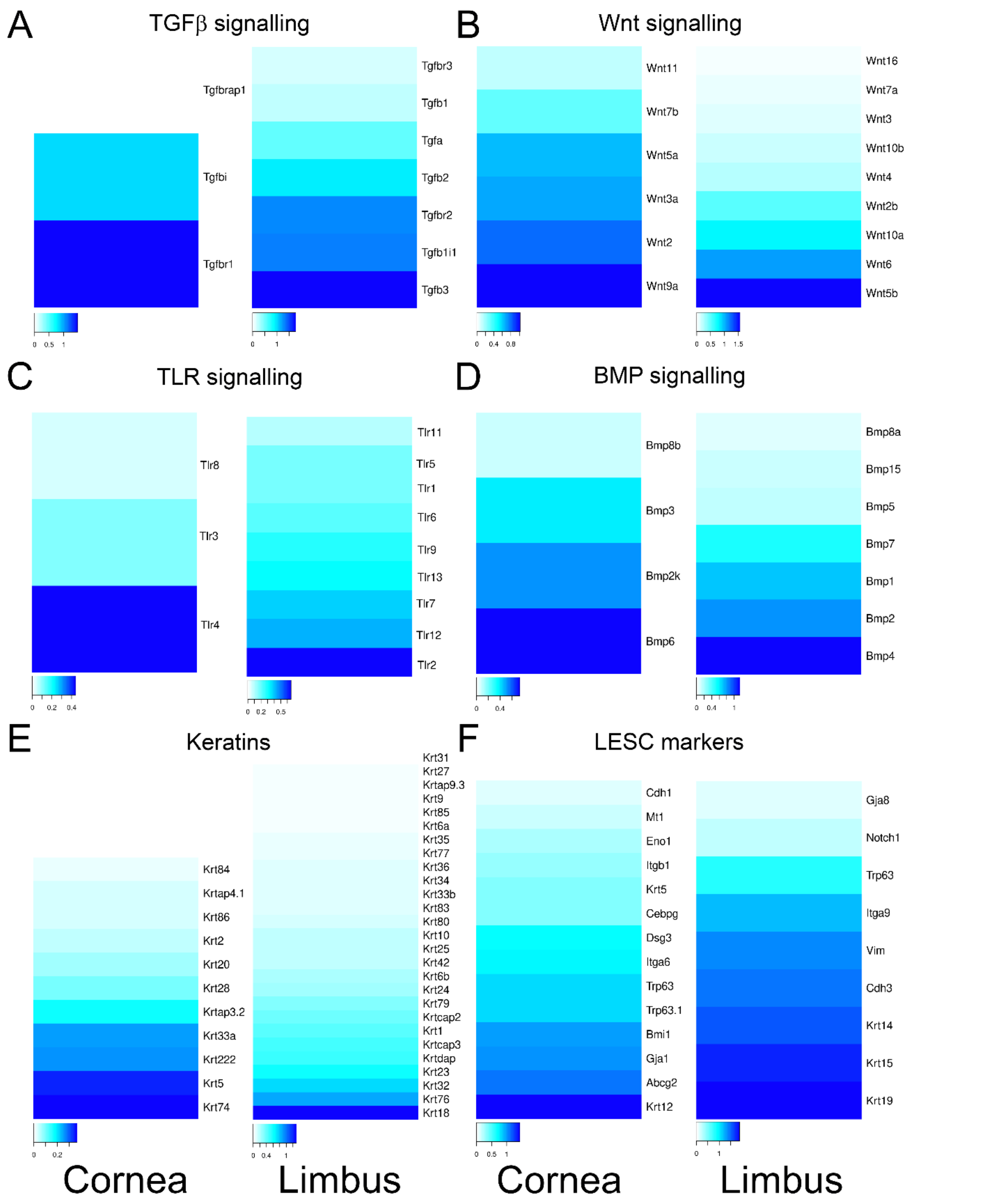

Differential expression of genes involved in signaling pathways and genes for putative stem cell markers between the central cornea and limbus in mice. Heatmaps were generated using

Heatmapper showing differential expression of genes between the central cornea and the limbus for TGF-ß signaling (A), Wnt signaling (B), Toll-like receptor signaling (C), bone morphogenic protein (BMP) signaling (D), keratins (E), and LESC markers (F). The heatmap representing the color-coded fold expression for each heatmap is shown underneath each heatmap. Darker blue colors represent a higher fold difference. Heatmaps to the left indicate genes that are upregulated in the central cornea when compared to the limbal region, and heatmaps to the right indicate genes that are upregulated in the limbus when compared to the central cornea.
