# Supplementary material for: Characterization of the Limbal Epithelial Stem Cell Niche
Source: Invest Ophthalmol Vis Sci. 2023 Oct 31;64(13):48. doi: 10.1167/iovs.64.13.48 (PMC10619699; doi:10.1167/iovs.64.13.48)
Supplement: Supplement 2 [file iovs-64-13-48_s002.pdf]

Supplementary Table 1

Table 1: Sequences of primers used for real time PCR

Gene	Accession number	Primers	Forward	Reverse
Chst10	NM_145142.2	1	GCCGATGTACGAGGACCAT	TTCCATCACGGACCATCTGC
		2	CGCCGATGTACGAGGACCAT	TCACATTGCCAAGCTGGACT
Itih5	NM_172471.2	1	CCTGCAGGATGCTCAACAGA	ACGCTGTCTCCTATAAGCATGG
		2	TATGCCTTCACCACGGTGTC	CGCTGTCTCCTATAAGCATGG
Itih2	NM_010582.3	1	TGCCTCAGAGTGTCGTGTTC	ATGCCGTTCACAGTCATGGT
		2	TCCTGAGAACCTGGACCCAA	GCCTCCACAGTCTGCTTCAT
Tgfb1	NM_011577.2	1	GCTGAACCAAGGAGACGGAA	ATGTCATGGATGGTGCCCAG
		2	CTGCTGACCCCCACTGATAC	AGCCCTGTATTCCGTCTCCT
Tgfb2	NM_001329107.1	1	CTGTGGGTACCTTGATGCCA	CTCTGGCTTTGGGGTTTTGC
		2	CTCCCCTCCGAAACTGTCTG	CTGTCTGGAGCAAAAGCTGC
Tgfb3	NM_009368.3	1	GAAGAGATGCACGGGGAGAG	CAGACGGCCAGTTCATTGTG
		2	TTACTGCTTCCGCAACCTGG	AGGTTCGTGGACCCATTTCC
Gapdh	NM_001289726.1	1	AACAGCAACTCCCACTCTTC	CCTGTTGCTGTAGCCGTATT
Actb	NM_007393.5	1	CACTGTCGAGTCGCGTCC	TCATCCATGGCGAACTGGTG
