# Supplementary material for: Characterization of the Limbal Epithelial Stem Cell Niche
Source: Invest Ophthalmol Vis Sci. 2023 Oct 31;64(13):48. doi: 10.1167/iovs.64.13.48 (PMC10619699; doi:10.1167/iovs.64.13.48)
Supplement: Supplement 3 [file iovs-64-13-48_s003.docx]

1. **This script allows for conversion of data from UCSC cell browser to Scanpy.**

###################################################################################

#Step 1. Choose your project at UCSC cell brownser. Go to Info > Data Download and#

#exprMatrix.tsv.gz and meta.tsv.###################################################

###################################################################################

#Step2. Use Seurat to convert barcodes and metadata to scanpy.#####################

require(Seurat)

require(data.table)

setwd("Project Name")

mat <- fread("exprMatrix.tsv.gz")

meta <- read.table("meta.tsv", header=T, sep="\t", as.is=T, row.names=1)

genes = mat[,1][[1]]

genes = gsub(".+[|]", "", genes)

mat = data.frame(mat[,-1], row.names=genes)

so <- CreateSeuratObject(counts = mat, project = "Project Name", meta.data=meta)

###################################################################################

#Step3. Save data as h5.Seurat

library(SeuratDisk)

SaveH5Seurat(so, filename = "pbmc3k.h5Seurat")

###################################################################################

#Step4. Convert seurat to anndata using sceasy

if (!requireNamespace("BiocManager", quietly = TRUE))

install.packages("BiocManager")

BiocManager::install(c("LoomExperiment", "SingleCellExperiment"))

library(sceasy)

library(reticulate)

conda_create("loompy")

conda_install(envname = "loompy",

packages = "loompy",

channel = "bioconda")

use_condaenv("loompy", required = TRUE)

py_config()

py_module_available("loompy")

use_condaenv('loompy')

loompy <- reticulate::import('loompy')

Convert("pbmc3k.h5Seurat", dest = "h5ad")

###################################################################################

#load data into scanpy

import scanpy as sc

import pandas as pd

from matplotlib import rcParams

sc.set_figure_params(dpi=80, color_map='viridis')

sc.settings.verbosity = 2

sc.logging.print_versions()

adata = sc.read("pbmc3k.h5ad", var_names='gene_symbols',cache=True)

adata.raw = adata

marker_genes = ['4323', 'LUM', 'DEC']

sc.set_figure_params(scanpy=True, dpi=300, dpi_save=300, frameon=True,

vector_friendly=True, fontsize=14, figsize='16,8', color_map=None,

format='tiff', facecolor=None, transparent=False)

ax = sc.pl.dotplot(adata, marker_genes, groupby='cluster',

cmap='viridis', save='Figure.tiff')

###################################################################################

1. **This sample script performs scanpy analysis**

###################################################################################

import numpy as np

import pandas as pd

import scanpy as sc

sc.settings.verbosity = 3

sc.logging.print_header()

sc.settings.set_figure_params(dpi=100, facecolor='white')

results_file = 'write/pbmc3k1.h5ad' # the file that will store the analysis results

adata = sc.read_csv(

'exprMatrix.tsv', # the directory with the `.mtx` file

delimiter=',',

dtype='float32')

adata.var_names_make_unique()

adata

adata.obs

#sc.pl.highest_expr_genes(adata, n_top=20, )

sc.pp.filter_cells(adata, min_genes=200)

sc.pp.filter_genes(adata, min_cells=10)

adata.var['mt'] = adata.var_names.str.startswith('MT-') # annotate the group of mitochondrial genes as 'mt'

sc.pp.calculate_qc_metrics(adata, qc_vars=['mt'], percent_top=None, log1p=False, inplace=True)

sc.pl.violin(adata, ['n_genes_by_counts', 'total_counts', 'pct_counts_mt'],

jitter=0.4, multi_panel=True)

#########################################################################################

sc.pl.scatter(adata, x='total_counts', y='pct_counts_mt')

sc.pl.scatter(adata, x='total_counts', y='n_genes_by_counts')

adata = adata[adata.obs.n_genes_by_counts < 2500, :]

adata = adata[adata.obs.pct_counts_mt < 5, :]

sc.pp.normalize_total(adata, target_sum=1e4)

sc.pp.log1p(adata)

sc.pp.highly_variable_genes(adata, min_mean=0.0125, max_mean=8, min_disp=0.5)

sc.pl.highly_variable_genes(adata)

###################################################################################

adata.raw = adata

adata = adata[:, adata.var.highly_variable]

sc.pp.regress_out(adata, ['total_counts', 'pct_counts_mt'])

sc.pp.scale(adata, max_value=10)

###################################################################################

sc.tl.pca(adata, svd_solver='arpack')

sc.pl.pca(adata, color='CST3')

sc.pl.pca_variance_ratio(adata, log=True)

adata.write(results_file)

adata

###################################################################################

sc.pp.neighbors(adata, n_neighbors=4, n_pcs=39)

#sc.pp.neighbors(adata, n_neighbors=6, n_pcs=24)

sc.tl.leiden(adata)

###################################################################################

sc.tl.paga(adata)

pl.paga(adata, plot=False) # remove `plot=False` if you want to see the coarse-grained graph

sc.tl.umap(adata, init_pos='paga')

sc.tl.umap(adata)

sc.pl.umap(adata, color=['CST3', 'NKG7', 'PPBP'], use_raw=False)

sc.tl.leiden(adata)

sc.pl.umap(adata, color=['leiden', 'CST3', 'NKG7'])

adata.write(results_file)

sc.tl.rank_genes_groups(adata, 'leiden', method='t-test')

sc.pl.rank_genes_groups(adata, n_genes=25, sharey=False, save='ttest.pdf')

sc.tl.rank_genes_groups(adata, 'leiden', method='wilcoxon')

sc.pl.rank_genes_groups(adata, n_genes=25, sharey=False, save='wilcoxon.pdf')

sc.tl.rank_genes_groups(adata, 'leiden', method='logreg')

sc.pl.rank_genes_groups(adata, n_genes=25, sharey=False, save='logreg.pdf')

marker_genes = ['HAS1', 'HAS2', 'HAS3', 'ITIH1', 'ITIH3', 'ITIH4',

'ITIH5', 'TNFAIP6', 'HYAL1', 'HYAL2', 'HYAL3',

'HYAL4', 'CD44', 'PTX3', 'VCAN', 'ACAN', 'KERA', 'LUM', 'DCN', 'BGN', 'FMOD']

new_cluster_names = ['Limbal Suprabasal Cells', 'Melanocytes', 'Corneal Superficial Epithelium', 'Corneal Endothelium', 'Blood Vessels', 'Conjunctival Superficial Epithelium',

'Corneal Basal Epithelium', 'Corneal stromal keratocytes','Lymphatic vessels', 'Limbal Progenitor cells','Limbal Superficial Epithelium',

'Limbal Stroma Keratocytes','Corneal Stromal Stem Cells', 'Conjunctival Basal Epithelium',

'Limbal Fibroblasts','Corneal Suprabasal Cells', 'Fibroblastic Corneal Endothelial Cells',

'Immune cells 2','Immune cells 1', 'Limbal Neural Crest Progenitors', 'Red Blood Cells']

new_cluster_names = ['0', '1', '2', '3', '4', '5','6', '7','8', '9','10','11','12', '13', '14','15', '16', '17', '18', '19', '20', '21' ]

adata.rename_categories('leiden', new_cluster_names)

fig = plt.figure(figsize=(6, 6))
